# Cryo-EM reveals ArnA contamination during purification of a ciliary protein complex

**DOI:** 10.1107/S2059798326002846

**Published:** 2026-04-15

**Authors:** Xuguang Jiang, Masahide Kikkawa

**Affiliations:** a Department of Cell Biology and Anatomy, Graduate School of Medicine The University of Tokyo Tokyo113-0033 Japan; University of Queensland, Australia

**Keywords:** cryo-electron microscopy, protein purification, *Escherichia coli* contaminants, ArnA, His-tag affinity chromatography

## Abstract

Endogenous *Escherichia coli* proteins can co-purify with recombinant targets and dominate cryo-EM datasets, yet often escape detection during standard biochemical quality control. In parallel to the recent report by Caliseki and coworkers [Caliseki *et al.* (2025), *Acta Cryst.* D**81**, 545–557], we independently identified ArnA contamination while purifying a soluble, low-yield KIF17–IFT70 complex, ultimately obtaining a 3.23 Å resolution cryo-EM structure of ArnA rather than the intended target. Our results reinforce that ArnA enrichment reflects general features of His-tag affinity purification and can become particularly problematic in cryo-EM workflows when the intended target is low in yield or conformationally heterogeneous. By comparing biochemical behavior and cryo-EM outcomes, we outline why ArnA may evade SDS–PAGE and size-exclusion chromatography, and be underappreciated in routine mass spectrometry-based quality control, yet becomes structurally dominant in cryo-EM. These findings broaden the scope of the original study and highlight the need for early cryo-EM screening and improved contaminant awareness in structural biology.

## Abstract 2

ArnA contamination was independently observed during the purification of a soluble, low-yield protein complex, leading to an unintended 3.23 Å resolution cryo-EM structure. This work broadens recent findings and highlights ArnA as a recurrent His-tag-associated contaminant in *Escherichia coli* purification workflows.

## 1. Introduction

The recent report by Caliseki and coworkers details an unexpected yet crucial observation: endogenous *Escherichia coli* proteins such as ArnA and AcrB can co-purify during His-tag affinity purification and subsequently overwhelm cryo-electron microscopy (cryo-EM) datasets when they are biochemically inconspicuous in SDS–PAGE or are overlooked in routine mass spectrometry (MS)-based quality control (QC) analyses (Caliseki *et al.*, 2025 ▸). Their work underscores a problem that has been systematically underestimated in cryo-EM structural analysis: stable endogenous complexes from expression hosts can credibly mimic intended targets and yield high-resolution contaminant structures.

It has long been recognized that His-tag purification from *E. coli* can lead to enrichment of histidine-rich host proteins (Wülfing *et al.*, 1994 ▸; Bolanos-Garcia & Davies, 2006 ▸). ArnA is among the most frequent and problematic of these contaminants, owing to its stability and its strong avidity for metal-chelating matrices (Williams *et al.*, 2005 ▸; Andersen *et al.*, 2013 ▸). Indeed, ArnA has been explicitly targeted for removal in engineered *E. coli* expression strains such as LOBSTR (Robichon *et al.*, 2011 ▸; Andersen *et al.*, 2013 ▸).

Although ArnA contamination has often been discussed in specific experimental contexts, its enrichment reflects general features of His-tag affinity purification rather than sample class (Caliseki *et al.*, 2025 ▸). Importantly, in cryo-EM workflows, rigid endogenous assemblies such as ArnA can disproportionately dominate datasets when the intended target is low in yield or conformationally heterogeneous.

Here, we present an independent observation that reinforces and broadens this conclusion. While attempting to determine the structure of a soluble full-length kinesin-2–cargo complex from *Caenorhabditis elegans*, KIF17–IFT70 (also known as OSM-3–DYF-1; Scholey, 2013 ▸; Mohamed *et al.*, 2018 ▸), we inadvertently solved a 3.23 Å resolution structure of ArnA, despite our target being an unrelated cytosolic assembly. Together with the report by Caliseki and coworkers, our observation highlights that well known His-tag-associated contaminants such as ArnA can disproportionately dominate cryo-EM datasets. This behavior reflects general properties of His-tag affinity purification combined with the strong particle-alignment advantage of rigid endogenous assemblies.

## 2. ArnA as a recurrent contaminant in His-tag affinity purification

**Figure 1:**
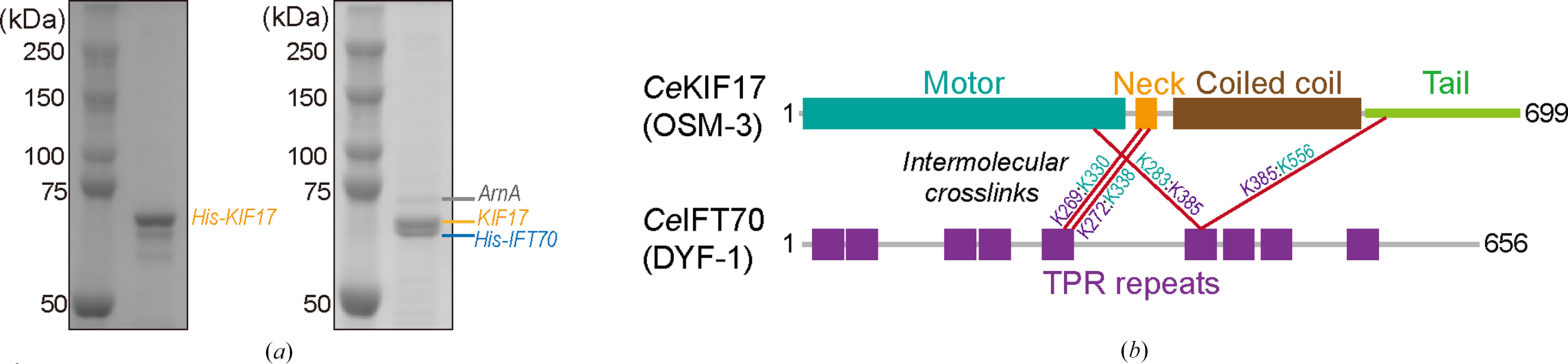
Biochemical characterization of the intended KIF17–IFT70 complex and concealed ArnA contamination. (*a*) SDS–PAGE analysis of concentrated samples following His-tag affinity purification and SEC. Lanes show purified His-KIF17 alone (left panel) and the reconstituted KIF17–His-IFT70 complex (right panel), allowing direct comparison of band positions with their theoretical molecular weights (MWs). The major bands corresponding to KIF17 and IFT70 were assigned based on this comparative purification under identical conditions. Upon contrast adjustment, an additional faint band migrating near ∼74 kDa can be discerned, consistent with the expected MW of ArnA and comparable to ArnA bands reported previously (see, for example, Shin *et al.*, 2025 ▸). Apparent MWs in SDS–PAGE deviate slightly from theoretical values, consistent with the elongated and partially disordered nature of KIF17 and IFT70. (*b*) Mass-spectrometry analysis of the purified sample. Four intermolecular cross-links are indicated by red lines. Cross-linking mass spectrometry confirmed the presence of KIF17 and IFT70 peptides and provided interaction information consistent with complex formation. ArnA peptides were not initially considered during data interpretation but were detected retrospectively upon re-analysis of the dataset.

Our work focused on purifying a soluble KIF17–IFT70 complex (∼235 kDa). Because IFT70 tends to aggregate and form inclusion bodies when expressed alone, we used a co-expression strategy that produced a seemingly clean preparation after His-tag affinity purification followed by size-exclusion chromatography (SEC). The final concentrated samples appeared to be biochemically clean by standard criteria. SDS–PAGE analysis showed dominant bands at positions consistent with the theoretical molecular weights (MWs) of KIF17 and IFT70, as confirmed by direct comparison with samples in which His-KIF17 was purified alone under identical conditions (Fig. 1 ▸*a*). No prominent additional bands were evident under standard staining conditions. Mass spectrometry confirmed the expected components and provided cross-linking information (Fig. 1 ▸*b*).

However, upon closer inspection and contrast adjustment, a weak band migrating near ∼74 kDa could be detected (Fig. 1 ▸*a*, right panel), consistent with the MW of ArnA and comparable to previously reported ArnA SDS–PAGE profiles (see, for example, Shin *et al.*, 2025 ▸). These observations illustrate how ArnA contamination can remain inconspicuous at the biochemical level while persisting through purification.

Despite these QC readouts appearing to be acceptable by standard biochemical criteria, the sample was not what it appeared. The yield of the KIF17–IFT70 complex remained low (∼0.2 mg per 4 l culture), and the complex displayed some aggregation during dialysis and ultrafiltration, features typical of assembly-prone IFT proteins. Such instability is expected to compromise the formation of self-consistent particle views in single-particle cryo-EM.

The theoretical MW of ArnA (∼74 kDa) overlaps substantially with those of KIF17 (∼79 kDa) and IFT70 (∼76 kDa) in SDS–PAGE. Notably, both KIF17 and IFT70 consistently migrated slightly faster than their theoretical MWs, appearing below the 75 kDa marker (Fig. 1 ▸*a*), a behavior commonly observed for elongated, coiled-coil- and IDR-rich motor and IFT proteins in SDS–PAGE. This apparent mobility shift may reflect a nonglobular protein architecture and, potentially, minor trimming of flexible regions, rather than incorrect protein assignment. Under these conditions, low-level ArnA contamination would be difficult to distinguish based on electrophoretic mobility alone.

In SEC, the expected broad elution range of the ArnA hexamer falls within the size window often associated with large soluble complexes or mild aggregation of the target. ArnA assembles into a stable hexamer with an approximate MW of ∼446 kDa, whereas the expected *C. elegans* KIF17–IFT70 complex has a MW of ∼235 kDa assuming a 2:1 stoichiometry. Although these values differ substantially, SEC separation is governed by hydrodynamic (Stokes) radius rather than MW alone. In our case, SEC was performed using a Superdex 200 Increase column, the resolving power of which decreases for large assemblies approaching its upper fractionation limit (∼600 kDa). Furthermore, the KIF17–IFT70 complex is expected to adopt an extended, nonglobular conformation, which would increase its apparent hydrodynamic radius and shift its elution toward earlier volumes. Under these conditions, partial overlap between the extended KIF17–IFT70 complex and the compact ArnA hexamer is plausible, complicating separation by SEC alone. As a result, ArnA was not clearly separated from the KIF17–IFT70 complex and could easily be misinterpreted as sample heterogeneity.

Moreover, ArnA is often detected by MS but with relatively low spectral counts in nonquantitative or target-focused analyses, particularly when not anticipated as a contaminant, rendering it prone to being overlooked or deprioritized during routine proteomics QC. Consistent with previous reports, retrospective inspection of our XL–MS datasets revealed ArnA-derived peptides (Abeyrathne & Grigorieff, 2017 ▸; Gomez-Raya-Vilanova *et al.*, 2022 ▸; Caliseki *et al.*, 2025 ▸), which were initially not emphasized as ArnA was not anticipated as a major contaminant at that stage. Importantly, these biochemical features alone do not fully explain the dominance of ArnA in cryo-EM datasets. These combined biochemical features explain how ArnA passed unimpeded through our purification and appeared deceptively benign. This issue becomes especially acute when the target protein is low in yield, as even a minor fraction of ArnA can represent a disproportionately large fraction of the high-quality, alignable particles during cryo-EM analysis.

## 3. Cryo-EM rapidly uncovers ArnA despite its biochemical subtlety in standard QC assays

**Figure 2:**
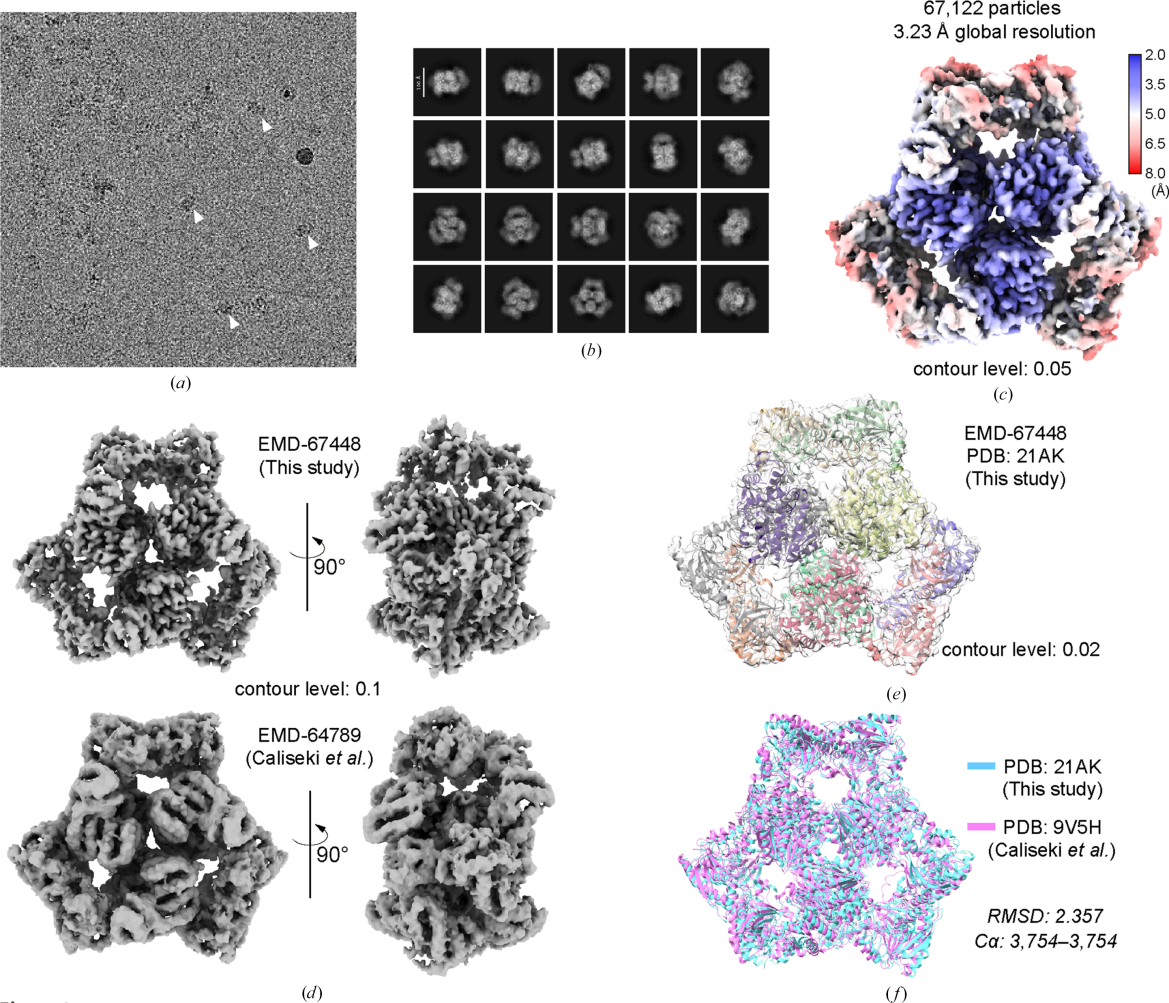
Cryo-EM identification and high-resolution structure determination of ArnA contaminant. (*a*) Representative motion-corrected cryo-EM micrograph. Cryo-EM screening revealed heterogeneous fields containing regions of aggregation alongside dispersed, homogeneous particles suitable for single-particle analysis. White arrows mark dispersed ArnA particles. (*b*) 2D classification of cryo-EM particles. Scale bar: 11 nm. Unexpectedly, 2D classification yielded highly homogeneous class averages exhibiting threefold-symmetric features characteristic of the ArnA hexamer, rather than views consistent with the intended KIF17–IFT70 complex. (*c*) Cryo-EM reconstruction of ArnA obtained from the dataset. *Ab initio* reconstruction and subsequent refinement produced a threefold-symmetric map corresponding to the ArnA hexamer, refined to an overall resolution of 3.23 Å. The resulting density is consistent with previously reported ArnA architectures, confirming that the dominant particles in the dataset correspond to ArnA rather than the intended target complex. The final reconstructed ArnA map and local resolution distribution are shown. (*d*) Comparison of the ArnA map obtained in this study with that reported by Caliseki and coworkers. (*e*) Superposition of the ArnA structural model determined here with the corresponding EM map, shown in transparent gray. (*f*) Structural superimposition of the ArnA models from this study and from that of Caliseki and coworkers.

Initial cryo-EM screening revealed micrographs containing both regions of protein aggregation and a subset of dispersed homogeneous particles (Fig. 2 ▸*a*). Notably, similar features were already apparent at the level of negative-stain EM screening, where rigid, homogeneous particles coexisted with more diffuse and heterogeneous material. To our surprise, 2D classification yielded clear threefold-symmetric views, characteristic of the ArnA hexamer (Fig. 2 ▸*b*). *Ab initio* reconstruction and heterogeneous refinement produced an ArnA-like map at ∼4 Å resolution, and subsequent refinement yielded a final 3.23 Å resolution structure with *D*3 symmetry (Fig. 2 ▸*c*), the architectural features of which are in strong agreement with previously reported ArnA models (Yang *et al.*, 2019 ▸; Caliseki *et al.*, 2025 ▸; Figs. 2 ▸*d*–2 ▸*f*). Crucially, no convincing classes corresponding to the intended KIF17–IFT70 complex were identified.

The cryo-EM behavior of the sample can be rationalized by several factors.

(i) ArnA assembles into a rigid, highly symmetric oligomer, which aligns efficiently and generates exceptionally clean 2D class averages.
(ii) The intended KIF17–IFT70 complex is inherently flexible, low in abundance and aggregation-prone, diminishing its ability to yield meaningful particles during classification.
(iii) Even if ArnA constitutes only a minor fraction of the total protein mass, its stability and homogeneity allow it to dominate cryo-EM reconstructions.

Importantly, these features can be diagnostic at the negative-stain stage, where rigid symmetric assemblies can be readily distinguished from flexible or heterogeneous targets, highlighting the value of negative-stain EM as an early decision point rather than a purely preparative step.

These observations are consistent with the mechanisms proposed by Caliseki and coworkers: biochemically elusive contaminants can become overwhelmingly dominant in single-particle cryo-EM, particularly when the intended target is heterogeneous or fragile.

## 4. Broader implications for protein purification and cryo-EM workflows

Our findings extend the conclusions of the original report and argue that ArnA contamination should be considered a general concern in His-tag purification for low-yield and unstable targets in cryo-EM.

Two points deserve emphasis.

**Figure 3:**
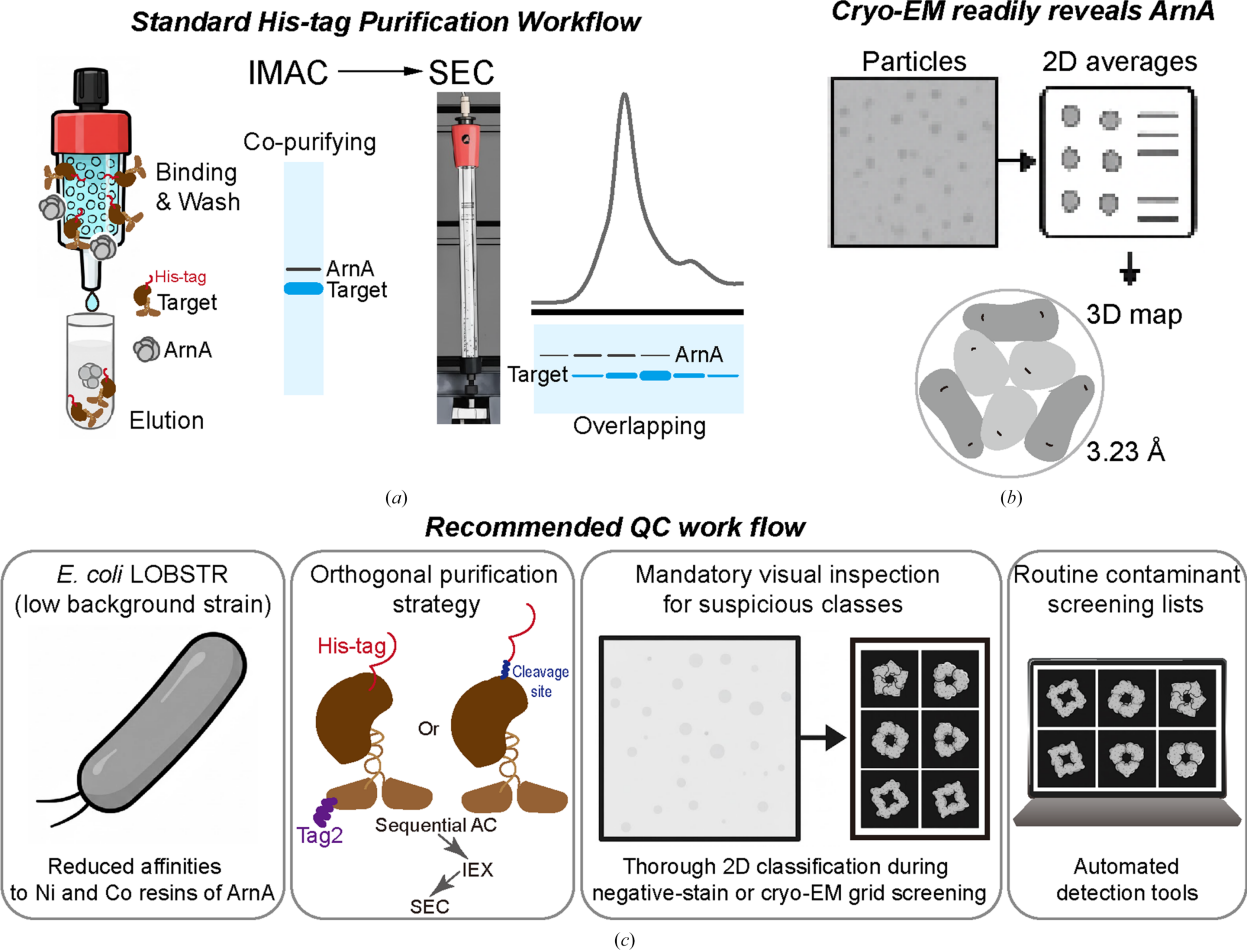
Mechanisms underlying the persistence of ArnA through standard QC and its impact on cryo-EM analysis, with practical mitigation strategies. (*a*) His-tag purification allows endogenous ArnA to co-purify. Schematic illustration of partial overlap between ArnA and the intended target during size-exclusion chromatography (SEC). ArnA assembles into a compact hexamer (∼446 kDa), while the expected *C. elegans* KIF17–IFT70 complex (∼235 kDa, assuming a 2:1 stoichiometry) is predicted to adopt an extended, nonglobular conformation. Because SEC separation depends on hydrodynamic (Stokes) radius rather than MW alone, the extended KIF17–IFT70 complex can exhibit an increased apparent size and elute earlier than expected. When using a Superdex 200 Increase column, the resolving power of which decreases near its upper fractionation range (∼600 kDa), this effect can lead to partial elution overlap between the ArnA hexamer and the target complex, complicating separation by SEC alone. All panels are schematic illustrations and do not represent experimental data. (*b*) Cryo-EM readily identifies ArnA via its symmetric hexameric particles. Schematic comparison of particle alignability between rigid endogenous assemblies and flexible target complexes in single-particle cryo-EM. ArnA forms a rigid, symmetric hexamer that readily yields high-contrast projections and clean 2D class averages, facilitating rapid alignment and reconstruction. In contrast, the intended KIF17–IFT70 complex is low-yield, conformationally flexible and prone to partial disorder, resulting in heterogeneous particle views that are less competitive during alignment and classification. Even when present at lower abundance, rigid endogenous assemblies such as ArnA can therefore disproportionately dominate cryo-EM datasets. All panels are schematic illustrations and do not represent experimental data. (*c*) Suggested QC steps for low-yield recombinant proteins. Suggested QC checkpoints to minimize contaminant-driven cryo-EM reconstructions. Standard biochemical QC steps (SDS–PAGE, SEC and MS) may be insufficient to flag rigid endogenous contaminants present at low abundance. Early cryo-EM screening, particularly negative-stain EM and small-scale grid screening followed by 2D classification, can rapidly reveal homogeneous, symmetric particles indicative of contaminants such as ArnA. Incorporating low-background expression strains, orthogonal purification strategies and early image-based QC can substantially reduce the risk of allocating extensive cryo-EM resources to unintended targets. All panels are schematic illustrations and do not represent experimental data.

(i) Soluble, low-yield complexes are particularly vulnerable. Such samples often produce deceptively clean SDS–PAGE profiles, and their inherent flexibility reduces their competitiveness during particle alignment (Fig. 3 ▸*a*). The symmetric hexameric architecture of ArnA, by contrast, readily outperforms them during 2D classification (Fig. 3 ▸*b*).
(ii) SEC alone may not reliably remove ArnA, depending on the size and conformation of the target complex and the resolving range of the column used. Because the ArnA hexamer (∼446 kDa) overlaps with the elution range of many biologically relevant multi-protein assemblies (Fig. 3 ▸*a*), it may persist through polishing steps unnoticed.

These considerations highlight a more general problem: standard biochemical QC (SDS–PAGE, SEC and routine MS-based analyses) can be insufficient for flagging endogenous rigid assemblies such as ArnA and AcrB. By contrast, early EM-based screening, particularly negative stain, directly probes particle homogeneity, rigidity and symmetry, providing information that is otherwise inaccessible to biochemical assays alone.

## 5. Practical recommendations for routine laboratory practice

To minimize the risk of contaminant-driven cryo-EM structures, we suggest the following pragmatic measures (Fig. 3 ▸*c*).

(i) Utilize low-background strains, such as *E. coli* LOBSTR, for His-tag purification (Andersen *et al.*, 2013 ▸).
(ii) Increase purification stringency by introducing orthogonal purification strategies. In addition to introducing alternative affinity tags, if the existing His-tag is cleavable, tag removal followed by a negative purification step, re-binding the cleaved sample to nickel resin and collecting the flowthrough, can be an effective way to remove His-rich endogenous contaminants, provided that cleavage is efficient. Ion-exchange chromatography represents another powerful orthogonal purification step, offering high binding capacity without the need to introduce additional affinity tags, and may further reduce co-purifying host proteins such as ArnA.
(iii) Examine early negative-stain grids for rigid, homogeneous symmetric particles. The presence of such assemblies should immediately raise suspicion of potential contaminants and should not be misinterpreted as improved sample integrity or successful complex formation.
(iv) Conduct thorough 2D classification during small-scale cryo-EM grid screening, including mandatory visual inspection for suspicious classes.
(v) Establish routine cryo-EM contaminant screening lists and consider developing and implementing automated detection tools such as *ASOCEM* (Eldar *et al.*, 2022 ▸) for characteristic symmetric assemblies during early image analysis. While ArnA and AcrB are used here as representative examples, numerous other recurrent host-derived contaminants have been documented, and curated databases developed in the crystallography community provide a more comprehensive reference set.

These steps can help prevent costly misallocation of microscope time and improve the reliability of structural workflows.

## 6. Discussion

The problem of co-purifying endogenous contaminants has long been appreciated in the protein crystallography community, where systematic efforts have led to curated contaminant databases and screening tools. For example, Janet Smith’s group recently reported the accidental crystallization of *E. coli* carbonic anhydrase II, a known contaminant (Rankin & Smith, 2025 ▸). In that case, the identification of new crystal forms further enabled their inclusion of its unit-cell parameters in search tools such as *SIMBAD* (Simpkin *et al.*, 2020 ▸). Moreover, databases such as ContaBase, accessed via tools including *ContaMiner*, are routinely consulted in crystallographic workflows to flag recurrent contaminants such as ArnA and AcrB (Hungler *et al.*, 2016 ▸). Importantly, proteins such as ArnA and AcrB were identified as frequent His-tag-associated contaminants independent of sample class well before the widespread adoption of cryo-EM, underscoring that their enrichment reflects general properties of affinity purification rather than unexpected sample-specific behavior.

Notably, recent cryo-EM studies explicitly acknowledge ArnA as a contaminant to be avoided during sample preparation, employing strategies such as multi-step purification (Shin *et al.*, 2025 ▸) or the use of engineered host strains including LOBSTR (Quail *et al.*, 2025 ▸; Tan *et al.*, 2025 ▸). These examples indicate growing awareness within the cryo-EM community. However, these studies also illustrate that awareness alone does not fully mitigate the problem, particularly when the intended target is low in yield or conformationally heterogeneous, conditions under which rigid endogenous assemblies can still dominate cryo-EM datasets.

Our independent identification of high-resolution ArnA contamination during the purification of a soluble, low-yield protein complex extends the conclusions of the original report and provides an additional cryo-EM-relevant case illustrating how ArnA, a well established His-rich *E. coli* contaminant, can dominate single-particle cryo-EM datasets when present alongside low-yield or heterogeneous targets. Despite its biochemical subtlety, ArnA can overwhelm cryo-EM datasets due to its stability and strong particle alignability. Recognizing this behavior and incorporating early cryo-EM screening into QC pipelines will help the structural biology community reduce wasted effort, avoid misinterpretation and ensure more reliable cryo-EM analyses.

Importantly, the present comment focuses on the methodological and workflow-related implications of contaminant enrichment in cryo-EM, rather than on the structural biology of ArnA itself. Although differences in relative domain orientations have been discussed in lower-resolution cryo-EM reconstructions (Yang *et al.*, 2019 ▸; Liu *et al.*, 2025 ▸) compared with available crystal structures (Gatzeva-Topalova *et al.*, 2005 ▸; Fischer *et al.*, 2015 ▸), the functional implications of these variations remain unclear and are beyond the scope of the present comment.

An open question raised by cases such as this is how unintended contaminant structures should be documented within the cryo-EM field. In macromolecular crystallography, such structures are often reported as short communications or technical notes, contributing to community-wide awareness and database development. Whether a similar practice should be adopted more systematically in cryo-EM remains an open discussion, but one that may help reduce redundant effort and improve early-stage sample assessment.

Moreover, beyond manually curated lists of known contaminants such as ArnA and AcrB, several efforts have begun to explore automated approaches to identifying characteristic assemblies in structural datasets. For example, recent work has proposed machine-assisted classification and detection methods that can flag symmetric or otherwise suspicious particle populations automatically (Eldar *et al.*, 2022 ▸). Moreover, workflows in proteomics regularly leverage curated contaminant databases and software to filter or annotate common background proteins, demonstrating how computational resources can be integrated into quality-control pipelines. While these tools are still evolving, their integration with early cryo-EM image analysis and other structural workflows may offer additional means to identify or exclude known contaminants in a systematic and scalable manner.

## 7. Materials and methods

### 7.1. Constructs, protein expression and purification

The genes encoding *C. elegans* KIF17 (OSM-3) and IFT70 (DYF-1) were synthesized with codon optimization and cloned into a pETDuet-1 vector with a His_6_-tag N-terminal to DYF-1. The KIF17-encoding gene was also cloned into a pET-28a vector with an N-terminal His_6_-tag for individual expression and purification. Recombinant plasmids were transformed into *E. coli* BL21 (DE3) (Novagen) and proteins were purified by His-tag affinity chromatography and SEC as described previously (Jiang *et al.*, 2025 ▸). Purified proteins were concentrated to 2 mg ml^−1^ using ultrafiltration, flash-frozen in liquid nitrogen and stored at −80°C.

### 7.2. Cryo-EM sample preparation, data collection and processing

3 µl of the sample at a concentration of 1 mg ml^−1^ was applied onto a hydrophilized holey carbon grid (Au, R1.2/1.3, 300 mesh, Quantifoil). The grid was then blotted for 4 s (blot force 10) and plunge-frozen in liquid ethane using a Vitrobot Mark IV (FEI) at 6°C and 100% humidity.

Images were acquired on a Titan Krios G3I (Thermo Fisher Scientific) microscope operating at 300 kV using *EPU* for automated data collection. Movie frames were recorded with a Gatan K3 direct electron detector operated in counting mode and correlated double-sampling (CDS) mode at a nominal magnification of 105 000×, yielding a pixel size of 0.65 Å per pixel. The data were collected in zero-loss mode with an energy filter slit width of 20 eV and an objective lens aperture diameter of 100 µm. 3000 movies were recorded with a total electron exposure of 61.6 e^−^ Å^−2^ over 63 frames. All data were processed using *CryoSPARC* (version 4.7.1; Punjani *et al.*, 2017 ▸). For particle picking, an initial blob picker was used to generate rough 2D templates, which were improved by 2D classification and then applied in the template picker for more accurate particle selection. Particles were first extracted with 4× binning (pixel size 2.6 Å; box size 249.6 Å) and subjected to several rounds of 2D classification (150 Å mask). High-quality classes were combined and de-duplicated, yielding 121 875 particles. All particles were used to generate three *ab initio* 3D models, which served as references for heterogeneous refinement to separate different conformational/compositional states. Selected classes were then re-extracted at 2× binning (1.3 Å) and refined using homogeneous and local refinement. CTF refinement and reference-based motion correction were performed before the final refinement. Summary statistics are provided in Supplementary Table S1.

The initial structural model was constructed by docking the previous PDB model (PDB entry 9v5h). The model was further refined using *Phenix* (Liebschner *et al.*, 2019 ▸), followed by manual adjustments in *Coot* (Emsley & Cowtan, 2004 ▸) to improve alignment with the EM density. A summary of model-building and refinement statistics is provided in Supplementary Table S1.

### 7.3. Cross-linking mass spectrometry

Protein samples were cross-linked using disuccinimidyl suberate (DSS; Dojindo) for 10 min at room temperature, followed by quenching with 50 m*M* Tris–HCl. The sample was then digested with trypsin, desalted and analyzed using a ZenoTOF7600 mass spectrometer (SCIEX) coupled with an UltiMate3000 RSLCnano system (Thermo Fisher Scientific). Cross-linked fragments containing DSS were analyzed using *MaxLynx* within the *MaxQuant* software suite. A summary of detected cross-links is provided in Supplementary Table S2.

## Supplementary Material

PDB reference: *E. coli* ArnA hexamer, 21ak

EMDB reference: *E. coli* ArnA hexamer, EMD-67448

Supplementary Tables S1 and S2. DOI: 10.1107/S2059798326002846/jb5071sup1.pdf

## Data Availability

Structural data have been deposited in the Electron Microscopy Data Bank (EMDB) as entry EMD-67448 (ArnA) and in the Protein Data Bank (PDB) as PDB entry 21ak. Other data are available in the main text or the supporting information.
