# Supplementary material for: Cryo-EM reveals ArnA contamination during purification of a ciliary protein complex
Source: Acta Crystallogr D Struct Biol. 2026 Apr 15;82(Pt 5):404–10. doi: 10.1107/S2059798326002846 (PMC13133990; doi:10.1107/S2059798326002846)
Supplement: Supplementary file 1 [file d-82-00404-sup1.pdf]

STRUCTURAL BIOLOGY

Volume 82 (2026)

Supporting information for article:

Cryo-EM reveals ArnA contamination during purification of a ciliary protein complex

Xuguang Jiang and Masahide Kikkawa

Table S1 Table S1. Cryo-EM data collection, modeling, and refinement statistics.

	E. coli **ArnA**<br>(EMD-67449; PDB: 21AK)
**Data collection and processing**	
Microscopy	Titan Krios G3i
Detector	Gatan K3
Voltage	300 kV
Cs (mm)	2.7
Slit width (eV)	20
Defocus range (μm)	0.8; 1.6
Electron exposure (*e/Å²*)	63
Pixel size (*Å*)	0.65
Fractions (no.)	63
Movies collected/used	3000/2979
Single particles (no.)	121,875
Single particles used (no.)	67,122
Overall resolution (*Å*)	3.23
FSC threshold	0.143
**Model building and refinement**	
Model composition	
Non-hydrogen atoms	28592
Protein residues	3612
R.m.s. deviation	
Bond lengths (*Å*)	0.004
Bond angles (°)	1.054
Validation	
MolProbity score	2.32
Clashscore	12.50
Poor rotamers (%)	3.86
Ramachandran plot	
Favored (%)	96.07
Allowed (%)	3.90
Outliers (%)	0.03

Table S2 Cross-links detected by cross-linking mass spectrometry.

Peptide	Peptide_Mass	Score	Crosslink type	Crosslinks
AELQKDLESLR(5)-SKHIPYR(2)	2339.27166	6.88E-01	Intra-Protein	OSM3(407)-OSM3(275)
DYQAVEKLFR(7)-YYWDKR(5)	2336.17089	2.76E-01	Intra-Protein	TTC30(451)-TTC30(443)
EQKTTDSDDSLEMR(3)-KTAIEIQIK(1)	2835.42906	7.58E-02	Intra-Protein	TTC30(405)-TTC30(393)
EQKTTDSDDSLEMR(3)-TAIEIQIKK(8)	2835.42906	6.79E-02	Intra-Protein	TTC30(405)-TTC30(401)
HVGATLMNKDSSR(9)-KSKGYER(1)	2420.23495	7.35E-01	Intra-Protein	OSM3(201)-OSM3(507)
KLGVDTWYYAK(1)-VVKALEPPEK(3)	2590.41659	8.46E-02	Intra-Protein	TTC30(584)-TTC30(576)
KQDQQLK(1)-AQSAKR(5)	1684.93398	6.88E-01	Intra-Protein	OSM3(535)-OSM3(688)
KSKGYER(3)-KSKGYER(1)	1871.9973	7.23E-01	Inter-Protein	OSM3(509)-OSM3(507)
KTAIEIQIK(1)-LEKECLDR(3)	2243.23144	4.29E-02	Intra-Protein	TTC30(393)-TTC30(385)
LEKLEKECLDR(6)-HIPYRDSKLTR(8)	2955.57191	6.05E-01	Inter-Protein	TTC30(385)-OSM3(283)
LEKLEKECLDR(6)-KDTNYSNVDR(1)	2781.37222	6.97E-01	Inter-Protein	TTC30(385)-OSM3(556)
LEKLEKECLDRLR(6)-LEKLEKECLDR(3)	3271.7387	7.32E-01	Intra-Protein	TTC30(385)-TTC30(382)
LGVDTWYYAKR(10)-ALEPPEKK(7)	2420.28591	2.81E-01	Intra-Protein	TTC30(594)-TTC30(583)
LSTSKSLFPSK(5)-AKNIKNK(5)	2147.24333	6.31E-01	Intra-Protein	OSM3(657)-OSM3(328)
MAESVRVAVRCR(1)-LASLNPK(7)	2313.25285	6.71E-01	Intra-Protein	OSM3(1)-OSM3(699)

NKPTINEDPK(2)-TKDFKAAK(5)	2201.18112	7.21E-01	Inter-Protein	OSM3(330)-TTC30(269)
NSVDYCKEHDTWK(7)-DYQAVEKLFR(7)	3087.45153	1.40E-01	Intra-Protein	TTC30(461)-TTC30(451)
PTINEDPKDALLR(8)-AAKESLTDMPPR(3)	2934.52396	6.92E-01	Inter-Protein	OSM3(338)-TTC30(272)
SKHIPYR(2)-YKSDQK(2)	1805.95438	7.08E-01	Intra-Protein	OSM3(275)-OSM3(648)
YANRAKNIKNK(9)-KDTNYSNVDR(1)	2668.38003	7.35E-01	Intra-Protein	OSM3(328)-OSM3(556)
YKDAAAFYEPIVHK(2)-LNVAHTIFMQEKK(12)	3347.74953	2.47E-01	Intra-Protein	TTC30(482)-TTC30(479)
